# Investigating the associations of the illness representations of gambling disorder with superstitious and responsible gambling

**DOI:** 10.3389/fpsyg.2023.1160781

**Published:** 2023-07-14

**Authors:** Anise M. S. Wu, Hong Mian Yang, Hui Zhou, Le Dang, Juliet Honglei Chen

**Affiliations:** ^1^Department of Psychology, Faculty of Social Sciences, University of Macau, Taipa, Macao SAR, China; ^2^Centre for Cognitive and Brain Sciences, Institute of Collaborative Innovation, University of Macau, Taipa, Macao SAR, China; ^3^Faculty of Teacher Education, Pingdingshan University, Pingdingshan, Henan, China; ^4^Department of Psychology, Zhejiang Sci-Tech University, Hangzhou, China

**Keywords:** commonsense model, illness perception, positive gambling, superstitious gambling, gambling beliefs

## Abstract

**Background:**

As a theoretical framework for understanding illness self-management, the commonsense model of self-regulation (CSM) has been commonly used to promote health behaviors. However, its application to examining gambling disorder (GD) is still in an exploratory stage.

**Objectives:**

Based on CSM, the current study aimed to address this knowledge gap and test whether illness representations (i.e., perceived consequences, illness coherence, and emotional representations) of GD are associated with gambling behaviors (i.e., responsible gambling [RG] and superstitious gambling). We also aimed to explore the potential mediating role of positive gambling beliefs (i.e., personal responsibility about gambling and gambling literacy) in such associations.

**Methods:**

An online questionnaire survey with snowballing sampling method was administered to Chinese adult past-year gamblers, and 603 valid responses were collected. The structural equation modeling (SEM) analysis with a bootstrapping approach was utilized to test the associations of illness representations with gambling behaviors and the hypothesized mediation effects of positive gambling beliefs.

**Results:**

We found that (a) perceived consequences of GD had significant, positive associations with RG and negative associations with superstitious gambling, with positive gambling beliefs acting as full mediators; (b) emotional representations for GD showed significant, negative correlations with RG and positive ones with superstitious gambling, with positive gambling beliefs acting as full and partial mediators, respectively; (c) the direct effect of illness coherence of GD on superstitious gambling behaviors was unexpectedly positive, and its indirect effects *via* positive gambling beliefs were nonsignificant.

**Discussion:**

Under the framework of CSM, the current findings provided new insights in understanding both controlled and at-risk gambling patterns from a perspective of illness self-management. We suggest future GD prevention campaigns may adopt psychoeducational programs to help gamblers form a better understanding about GD as an illness, which may promote RG practices and hence lower the risk of developing GD.

## Introduction

Gambling disorder (GD) is a serious behavioral addictive disorder that poses great harm to gamblers’ physical and mental health, as well as their social functioning (American Psychiatric Association, 2013; Langham et al., 2015; Abbott, 2020). A variety of risk and protective factors of GD have been investigated to inform prevention efforts to mitigate the development of GD; however, the results are still inconclusive (Menchon et al., 2018). To advance the current knowledge of GD, this study aimed to apply the common-sense model of self-regulation (CSM; Diefenbach and Leventhal, 1996; Leventhal et al., 2012, 2016) to gain a better understanding of individual differences regarding engagement in responsible and superstitious gambling to provide insights for GD-prevention efforts. According to CSM, individuals are active problem solvers in dealing with ongoing or future health threats (Leventhal et al., 1998). Based on external information and internal experiences, individuals form illness representations of a given illness, which determines their subsequent cognitive, affective, and behavioral responses to the health threat (Diefenbach and Leventhal, 1996; Leventhal et al., 1998). These illness representations are some common-sense beliefs about a certain illness and include a series of aspects, such as its cause (i.e., the cause of an illness), consequences (i.e., the perceived consequences of a health threat), illness coherence (i.e., the clear and coherent comprehension of an illness), and emotional representations (i.e., emotional responses to the illness; Moss-Morris et al., 2002).

In the past, CSM has been widely applied to physical diseases to facilitate patients’ health behaviors, such as treatment seeking and adherence (Hagger and Orbell, 2003), and these applications generally have shown considerable efficacy in improving individuals’ self-management in the face of health threats (Brandes and Mullan, 2014). However, only one published study to date has applied CSM to GD. Dang et al. (2022) adapted the Revised-Illness Perception Questionnaire (IPQ-R) to investigate GD among Chinese adults and found that compared to the non-GD group, the probable GD group reported lower perceived consequences of GD and showed stronger negative emotional responses to GD. They also found that ever-gamblers understood GD better than never-gamblers in that they had higher levels of illness coherence of GD. Their findings generally provided evidence that illness representations of GD are associated with individuals’ gambling engagement and patterns. If these associations can be replicated and extended to different patterns of gambling to improve our understanding of the underlying mechanism, then these representations may be targeted in future interventions for gambling problems. The present study hence aimed to explore the cognitive mechanisms of gambling behaviors (related to responsible gambling [RG] and superstitious gambling in our case) under the framework of CSM in a sample of Chinese adult gamblers; it tested (a) the associations between three types of illness representations of GD (i.e., perceived consequences, illness coherence, and emotional representations) and gambling behaviors, as well as (b) the potential mediation effects of positive gambling beliefs on these associations.

### Associations of illness representations of GD with RG and superstitious gambling

RG behaviors, from the perspective of gamblers, refer to the practices that an individual gambler implements to minimize the personal harm caused by gambling engagement (Blaszczynski et al., 2011), which is often manifested as setting time/money limits spent on gambling (i.e., precommitment to gambling involvement) and/or being honest about and take good control of gambling behaviors (i.e., honesty and control over gambling; Wood et al., 2017; Tong et al., 2020). Previous research has shown that RG behaviors are associated with a lower risk of GD and can be regarded as a GD-prevention strategy (Wood et al., 2017; Tong et al., 2020). In contrast, gambling-related superstitions appear to be manifestations of gambling-related cognitive distortions and are linked to higher levels of gambling intensity, as well as a higher risk of problem gambling (Joukhador et al., 2004; Wu et al., 2012; Leonard et al., 2015). In particular, superstitious gambling behaviors (e.g., wearing red underwear when gambling) have been shown to be positively correlated with symptoms of problem gambling in Chinese gamblers (Ohtsuka and Chan, 2010; Wu et al., 2012), who tend to be more likely to report these behaviors than gamblers from other countries (Kim et al., 2016; Chan et al., 2019). Under the theoretical framework of CSM, illness representations of GD and their associations with these two types of gambling behaviors were explored in this study, with the hope of discovering new ways to lower gamblers’ GD vulnerability and promote RG.

Gambling habits and behaviors are controllable for individuals who are not yet addicted to gambling (Currie et al., 2020). According to Dang et al.’s (Dang et al., 2022) pioneering study on illness representations of GD, people’s gambling behaviors vary according to their perceptions of the consequences of GD, perceived coherence of GD, and emotional representations of GD. In particular, gamblers of high GD risks were found to perceive GD as having fewer harmful consequences than their counterparts. Previous research also suggested that perceiving an illness as having severe consequences can have positive effects on individuals’ functioning and wellbeing by promoting individuals’ proactive coping (Hagger and Orbell, 2021), and Dang et al.’s finding is in keeping with the notion that gamblers who perceive GD as having severe adverse consequences would try to prevent themselves from developing GD and gamble in a more controlled manner in the first place. In this study, we hence made the following hypotheses:

*H1*a: Perceived consequences of GD is positively associated with RG behaviors.

*H1*b: Perceived consequences of GD is negatively associated with superstitious gambling behaviors.

Despite the scarcity of research on illness representations of GD, CSM has already been used to investigate various physical illnesses (e.g., breast cancer) and mental disorders (e.g., social anxiety disorder; Costanzo et al., 2011; Dias et al., 2018). Results of a recent meta-analysis of 254 studies based on CSM showed that among patients with chronic illnesses, illness coherence was positively associated with adaptive coping, better physical functioning, and psychological wellbeing (Hagger et al., 2017). In contrast, strong negative emotional representations of an illness were linked to a higher tendency to adopt avoidance coping strategies and negative health outcomes (Hagger et al., 2017). Given that a better understanding of mental disorders has been linked to higher levels of self-efficacy among patients with mental disorders (Goyal et al., 2020), it is plausible that perceived coherence of an illness may empower individuals to constructively manage their illness condition and to adopt positive strategies in coping with the illness. Taking GD as an example, gamblers with a clearer concept of GD (i.e., a higher level of illness coherence of GD) may feel more self-efficacious over controlling their gambling *via* RG practices and be less likely to engage in superstitious gambling, which is often done in an attempt to gain a sense of control *via* external means. Hence, we made the following hypotheses:

*H2*a: Illness coherence of GD is positively associated with RG behaviors.

*H2*b: Illness coherence of GD is negatively linked to superstitious gambling.

Conversely, individuals with strong negative emotional responses to a health threat are likely to become rapidly overwhelmed by negative emotions, which would take up lot of their resources for regulation and thus emotional-focused or avoidance coping strategy instead of a problem-focused coping strategy (Leventhal et al., 2016). Therefore, gamblers with high levels of emotional representations of GD (e.g., feeling very anxious and scared of GD) may tend to avoid thinking about GD-related problems and be less driven to control their gambling. Indeed, the only study on illness representations of GD has shown that probable GD gamblers tended to report stronger emotional representations of GD than their non-GD counterparts (Dang et al., 2022). In this study, we therefore made the following hypotheses:

*H3*a: Emotional representations of GD is associated with fewer RG behaviors.

*H3*b: Emotional representations of GD is associated with more superstitious gambling behaviors.

### Positive gambling beliefs as mediators

According to CSM, illness representations influence illness-related cognitions and emotions, which in turn determine one’s coping strategy and behaviors in response to the health threat (Leventhal et al., 1998). Along these lines, the cognitive-behavioral theory of problem gambling deems that gambling-related cognitions (e.g., gamblers’ beliefs regarding gambling outcomes) are a salient antecedent of gambling behaviors (Sharpe and Tarrier, 1993); moreover, evidence of this gambling-specific cognitive-behavioral link has been documented by an abundance of empirical research (Tang and Wu, 2012; Wu et al., 2013). For example, Goodie and Fortune (Goodie and Fortune, 2013) conducted a review and meta-analysis of studies using gambling-related beliefs scales, discovering that the effects of erroneous beliefs (e.g., illusion of control on game outcomes) on problem gambling were robust. On the other hand, accurate gambling beliefs were found to be positively associated with RG behaviors and negatively associated with GD symptoms (He et al., 2023).

According to both CSM and the cognitive-behavioral theory of problem gambling, gambling-specific cognitions (i.e., positive gambling beliefs in this study) is an antecedent of individuals’ gambling behaviors. Positive gambling beliefs refer to the beliefs about gambling that reduce gamblers’ risk for problem gambling (Wood et al., 2017). There are two major aspects of positive gambling beliefs, namely, personal responsibility (i.e., the belief that gamblers should take responsibility for not letting themselves fall into problem gambling) and gambling literacy (i.e., the recognition that gambling is not a way to make money and the awareness of the chance nature of gambling outcomes). If gamblers believe they are responsible for their gambling behaviors/outcomes and have an accurate understanding of the nature of games, they are more likely to gamble in a rational and responsible manner. Indeed, empirical findings have supported this premise: positive gambling beliefs have been shown to have significant, negative associations with gambling-related superstitions and positive associations with RG behaviors (Tong et al., 2020).

Research on GD illness representations and gambling-specific beliefs appears to be lacking. To our best knowledge, although CSM suggest that illness representations of GD may shape gamblers’ gambling beliefs, which in turn determine their gambling behaviors, no research to date has examined the indirect effects (e.g., *via* positive gambling beliefs in our case) of GD illness representations on gambling. This study was the first to empirically test the indirect role of illness representations of GD *via* the two mediators of positive gambling beliefs (i.e., personal responsibility and gambling literacy) on both responsible and superstitious gambling behaviors in order to clarify the cognitive mechanism underlying the effects of illness representations of GD on gambling patterns.

According to CSM, the negative association between perceived consequences and symptoms of GD (Dang et al., 2022) may be attributed to the motivational effect of the illness representation (Diefenbach and Leventhal, 1996; Cameron and Moss-Moris, 2010), which may drive gamblers to not only build greater awareness of their relation to, and responsibility over, the illness, but also to acquire better knowledge about games and gambling to avoid GD development; this resultant awareness and acknowledgment, in turn, are believed to result in more RG behaviors and fewer superstitious gambling behaviors. Therefore, it is hypothesized that:

*H4*a: Personal responsibility mediates (at least partially) the relationship between perceived consequences of GD and RG behaviors.

*H4*b: Gambling literacy mediates the relationship between perceived consequences of GD and RG behaviors.

*H4*c: Personal responsibility mediates the relationship between perceived consequences of GD and superstitious gambling.

*H4*d: Gambling literacy mediates the relationship between perceived consequences of GD and superstitious gambling.

Similarly, greater overall understanding of GD, which gamblers with high levels of illness coherence of GD have, may allow them to assume responsibility for their gambling behaviors and motivate them to improve their gambling literacy. We hence hypothesized that:

*H5*a: Personal responsibility mediates the association between illness coherence of GD and RG behaviors.

*H5*b: Gambling literacy mediates the association between illness coherence of GD and RG behaviors.

*H5*c: Personal responsibility mediates the association between illness coherence of GD and superstitious gambling.

*H5*d: Gambling literacy mediates the association between illness coherence of GD and superstitious gambling.

In contrast, stronger negative emotional responses to GD may consume the cognitive resources necessary to conduct a logical and thorough analysis of their responsibility as gamblers, as well as gambling rules or strategies; as a result, negative emotional responses to GD would be expected to lead to fewer RG behaviors and more superstitious gambling behaviors. In this study, we thus made the following hypotheses:

*H6*a: Personal responsibility mediates the association of emotional representations of GD with RG behaviors.

*H6*b: Gambling literacy mediates the association of emotional representations of GD with RG behaviors.

*H6*c: Personal responsibility mediates the association of emotional representations of GD with superstitious gambling.

*H6*d: Gambling literacy mediates the association of emotional representations of GD with superstitious gambling.

## Methods

### Participants and procedures

An online survey was conducted from February 2022 to March 2022. Ethics approval for this study was obtained from the department of psychology at the university to which the corresponding author is affiliated. A convenience sampling method *via* snowballing was adopted to recruit eligible participants who were required to be gamblers of Chinese ethnicity, aged 18 or above, who had engaged in gambling during the past 12 months. The questionnaire was written in simplified Chinese, and participation was completely anonymous and voluntary. The desired minimum sample size is determined as 330 according to the *N*:*p* ratio of 10:1 (*N* = the number of participants, *p* = the number of measured indicator variables; Nunnally, 1967). To encourage potential participants to actively take part in the study, those who completed the online questionnaire received a small but random amount of money as a reward (1–20 RMB [approximately 0.15–2.9 USD]). After reading the aim of the study and the rights of participants, as well as providing their consent to participate, participants began completing the formal questionnaire. In the end, a total of 603 valid responses were collected and included for formal analyses after three cases were excluded because of either a specific response pattern (i.e., selecting the first option on every item of all the eight [sub]scales of the current study) or missing all the items of the two dependent variables (i.e., RG behaviors and superstitious gambling behaviors). The characteristics of this sample are summarized in the section of *Sample Characteristics and Descriptive Analyses* in the Results section.

### Measures

#### Illness representations for GD

Three illness representations (i.e., consequences, illness coherence, and emotional representations) for GD were measured using the Chinese version of the Revised Illness Perceptions Scale for Gambling Disorder (Dang et al., 2022), which was validated among Chinese adults and showed satisfactory validity and reliability. Respondents rated agreement with items on a 5-point Likert scale (scores ranged from 1 to 5; 1 = *strongly disagree* and 5 = *strongly agree*), with higher mean scores representing higher corresponding illness representations of GD. For the 6-item consequences subscale (sample item “GD has major consequences on one’s life”), Cronbach’s α = 0.85 in this study. For the 5-item illness coherence subscale (sample item “I have a clear picture or understanding of GD”), α = 0.67. For the 5-item emotional representations subscale (sample item “You get depressed when you think about GD”), α = 0.91 in the current study.

#### Positive gambling beliefs

The positive play belief subscale of the Chinese version of the Positive Play Scale (PPS) was adopted to assess positive gambling beliefs (Wood et al., 2017; Tong et al., 2020). This scale consists of two dimensions, namely “personal responsibility” (4 items; sample item “I should only gamble when I have enough money to cover all my bills first”) and “gambling literacy” (3 items; sample item “Gambling is not a good way to make money”). Participants responded on a 5-point Likert scale in which 1 = *strongly disagree* and 5 = *strongly agree*, with a higher mean scale score representing a higher level of the corresponding belief. Given that previous validation studies have consistently found gambling literacy to have a relatively low α, based on their recommendations regarding this issue (Tabri et al., 2019; Tong et al., 2020), values of both Cronbach’s α and McDonald’s ω were computed to evaluate the reliability for the two subscales: for personal responsibility, α = 0.82, ω =0.89; and for gambling literacy, α = 0.56, ω =0.78, in the current study.

#### RG behaviors

The behavior subscale of the validated Chinese version of PPS was adopted to assess gamblers’ RG behaviors (Tong et al., 2020). This scale consists of two dimensions, namely RG-honesty and control (3 items; sample item “I was honest with my family and/or friends about the amount of time I spent gambling”) and RG-precommitment” (4 items; sample item “I considered the amount of money I was willing to lose before I gambled”), with both having a 5-point Likert response scale, in which 1 = *never* and 5 = *always*. A higher mean score represented a higher frequency of the corresponding type of RG behavior. For RG-honesty and control and RG-precommitment, α = 0.82 and 0.89, respectively, in our study.

#### Superstitious gambling behaviors

The superstition subscale of the behavior scale of the Revised Gambling Motives, Attitudes, and Behaviors Inventory (GMAB-R) was used to assess participants’ superstitious gambling behaviors (Wu et al., 2012). The subscale has three items, with a sample item being, “You gather charms to enhance your chance of winning.” Participants rated their agreement with these items on a 4-point Likert scale, in which 1 = *never* and 4 = always, in which higher mean scores represented higher frequencies of superstitious behaviors. For this subscale, α =0.70 in the current study.

### Demographics

Participants were asked to report their age (years), sex (male = 1, female = 2), and educational level (none = 1, primary = 2, junior high = 3, senior high = 4, undergraduate = 5, postgraduate = 6).

### Statistical analysis

Descriptive statistics and reliability analyses were conducted in SPSS 26.0. SEM, including measurement model testing and structural model testing, was conducted in Mplus 8.3 to determine how the hypothesized structural model for RG behaviors and superstitious gambling behaviors fit with our collected data. The full-information maximum likelihood (FIML) estimation was applied to handle missing values (Enders, 2010). Based on potential demographic effects on gambling cognitions/behaviors (Miller and Currie, 2008; Jimenez-Murcia et al., 2020; Allami et al., 2021), the three demographic variables (sex, age, and educational level) were controlled for in the structural model tested. As suggested (Hu and Bentler, 1999), comparative fit index (CFI; acceptable >0.90), Tucker-Lewis index (TLI; acceptable >0.90), standardized root mean square residual (SRMR; acceptable <0.08), and root mean square error of approximation (RMSEA; acceptable <0.08) were adopted to evaluate the goodness of model fit. For mediation effects testing, the indirect effects of illness representations for GD, *via* positive gambling beliefs to gambling behaviors, were examined using a bootstrapping approach with 5,000 re-samples in Mplus 8.3.

## Results

### Sample characteristics and descriptive analyses

Our sample consisted of 603 Chinese adult past-year gamblers (54.1% males), with a mean age of 40.57 years (*SD* = 12.11; range = 18–72 years). Around two thirds (i.e., 68.2%) of the participants had a college education or above, whereas 28.8 and 3.0%, respectively, received secondary education and primary education or below. As shown in Table 1, our participants were quite neutral when asked about their perceived coherence and emotional representations of GD (*M* = 2.82 and 3.08, respectively). In general, they tended to view GD as having severe consequences (*M* = 4.03) and to endorse beliefs about both gambling literacy (*M* = 3.93) and personal responsibility (*M* = 4.07). In addition, they reported more RG behaviors (*M* = 3.54 and 3.92 in honesty and control, as well as precommitment behaviors, respectively) but fewer superstitious behaviors (*M* = 1.58).

**Table 1:** Descriptive statistics for all measures (*N* = 603).

Measures	M	SD	Range
IR of GD - Consequences	4.03	0.76	1–5
IR of GD - Illness coherence	2.82	0.74	1–5
IR of GD - Emotional representations	3.08	0.92	1–5
Belief - Personal responsibility	4.07	0.79	1–5
Belief - Gambling literacy	3.93	0.76	1–5
RG-Honesty and control	3.54	1.22	1–5
RG-Precommitment	3.92	1.18	1–5
Superstitious gambling behaviors	1.58	0.61	1–4

M, mean of all items; SD, standard deviation; IR, illness representations; GD, gambling disorder; RG, responsible gambling.

### Measurement model

We first evaluated the goodness-of-fit of the original measurement model: *χ^2^*(467) = 1736.47, *p* < 0.001, *CFI* = 0.88, *TLI* = 0.86, *SRMR* = 0.08, and *RMSEA* = 0.07 (*90% CI* [0.064, 0.071]). The fit was only marginally satisfactory, and thus we added four pairs of within-variable residual covariances (i.e., residual covariance between item 5 and item 6 of consequences subscale; residual covariance between item 1 and item 2 of emotional representations subscale; residual covariance between item 1 and item 2, as well as between item 3 and item 4, of RG-precommitment subscale) based on the modification indexes. This procedure improved model fit to an acceptable level: *χ^2^*(463) = 1162.92, *p* < 0.001, *CFI* = 0.93, *TLI* = 0.92, *SRMR* = 0.08, and *RMSEA* = 0.05 (*90% CI* [0.046, 0.054]). The standardized factor loadings of all indicators for latent variables were also significant. Therefore, we were able to conclude that our measurement model fit the data well and was appropriate for structural modeling. The intercorrelation coefficients of all latent variables are shown in Table 2.

**Table 2:** Estimated correlation matrix for the latent variables (*N* = 603).

Latent variables	1	2	3	4	5	6	7
IR of GD - Consequences	–						
IR of GD - Illness coherence	−0.15^**^	–					
IR of GD - Emotional representations	0.16^***^	−0.28^***^	–				
Belief - Personal responsibility	0.55^***^	−0.03	−0.03	–			
Belief - Gambling literacy	0.27^***^	0.05	−0.17^***^	0.27^***^	–		
RG-Honesty and control	0.33^***^	0.10^*^	−0.12^*^	0.54^***^	0.26^***^	–	
RG-Precommitment	0.39^***^	0.07	−0.05	0.69^***^	0.22^***^	0.75^***^	–
Superstitious gambling behaviors	−0.14^**^	0.10	0.17^***^	−0.19^***^	−0.42^***^	−0.14^**^	0.00

IR, illness representation; GD, gambling disorder; RG, responsible gambling. ^*^p < 0.05, ^**^p < 0.01, ^***^p < 0.001.

### Structural model

**Figure 1:**
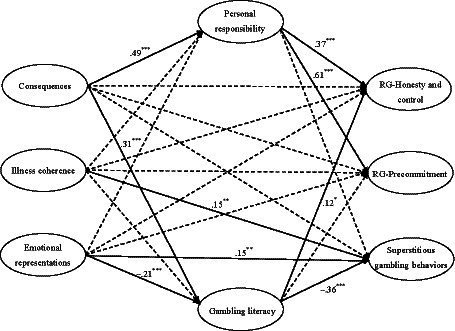
The structural model for responsible gambling behaviors and superstitious gambling. Sex, age, and educational levels were controlled for all latent variables in the model. Standardized coefficients are presented. Residuals covariance between RG-honesty and control and RG-precommitment is not shown. Coefficients of non-significant paths (dotted-line) (range from –0.06 to 0.08) are also not shown to keep the clarity of the figure. *p < 0.05, **p < 0.01, ***p < 0.001; RG, responsible gambling.

The structural model (see Figure 1) showed a good model fit, *χ^2^*(544) = 1414.78, *p* < 0.001, *CFI* = 0.92, *TLI* = 0.91, *SRMR* = 0.08, and *RMSEA* = 0.05 (*90% CI* [0.048, 0.055]). The model explained 39.9% of the variance in beliefs regarding personal responsibility, 15.9% in beliefs regarding gambling literacy, 38.6% in RG-honesty and control, 52.0% in RG-precommitment, and 24.1% in superstitious gambling behaviors.

As shown in Table 3 [(a) Total effects], the total effect of perceived consequences on the three behavioral constructs was statistically significant in the expected directions (*β* = 0.26 and 0.33, *p* < 0.001 for two types of RG behaviors and *β* = −0.17, *p* = 0.002 for superstitious behaviors), supporting *H1*a and *H1*b. However, the *H2*a and *H2*b hypotheses regarding illness coherence were not supported as both its total and direct effects on the two types of RG behaviors were nonsignificant (*p* = 0.056–0.188) while the total effect of illness coherence on superstitious behaviors was significant but positive (*β* = 0.15, *p* = 0.017). For emotional representations, its total effects on RG-honesty and control (but not RG-precommitment), as well as superstitious gambling behaviors, were statistically significant in the expected directions (*β* = −0.09, *p* = 0.039 and *β* = 0.23, *p* < 0.001, respectively), supporting *H3*a and *H3*b. As for the two positive gambling beliefs, belief in personal responsibility showed significant, positive associations to both perceived consequences of GD, as well as the two types of RG behaviors (*β* = 0.37–0.61, *p* < 0.001). Gambling literacy belief showed significant, negative links to emotional representations, as well as superstitious behaviors (*β* = −0.21 and − 0.36, *p* < 0.001), whereas it was positively linked to perceived consequences and RG-honesty and control (*β* = 0.31, *p* < 0.001 and *β* = 0.12, *p* = 0.013 respectively). However, illness coherence of GD was not significantly associated with any positive gambling beliefs and the indirect effects of illness coherence on RG behaviors, as well as superstitious gambling behaviors, were also nonsignificant. So, *H5*a, *H5*b, *H5*c, and *H5*d lacked support from our data.

**Table 3:** Testing the pathways of the mediation model (*N* = 603).

Path	Standardized effect	95% Confidence interval	
		Lower	Upper
**a. Total effects**			
Consequences → RG-Honesty and control	0.26^***^	0.173	0.352
Consequences → RG-Precommitment	0.33^***^	0.226	0.427
Consequences → Superstitious behaviors	−0.17^**^	−0.278	−0.062
Illness coherence → RG-Honesty and control	0.06	−0.031	0.153
Illness coherence → RG-Precommitment	0.07	−0.022	0.158
Illness coherence → Superstitious behaviors	0.15^*^	0.020	0.262
Emotional representations → RG-Honesty and control	−0.09^*^	−0.167	−0.004
Emotional representations → RG-Precommitment	−0.04	−0.126	0.042
Emotional representations → Superstitious behaviors	0.23^***^	0.126	0.323
**b. Mediation model**			
*Direct effects*			
Consequences → RG-Honesty and control	0.04	−0.069	0.152
Consequences → RG-Precommitment	0.01	−0.091	0.121
Consequences → Superstitious behaviors	−0.01	−0.133	0.127
Illness coherence → RG-Honesty and control	0.06	−0.020	0.149
Illness coherence → RG-Precommitment	0.08	−0.001	0.156
Illness coherence → Superstitious behaviors	0.15^**^	0.030	0.269
Emotional representations → RG-Honesty and control	−0.04	−0.121	0.044
Emotional representations → RG-Precommitment	0.00	−0.078	0.085
Emotional representations → Superstitious behaviors	0.15^**^	0.041	0.246
*Indirect effects: Positive ambling belief - Personal responsibility as the mediator*			
Consequences → Belief - Personal responsibility → RG-Honesty and control	0.19^***^	0.119	0.267
Consequences → Belief - Personal responsibility → RG-Precommitment	0.30^***^	0.218	0.400
Consequences → Belief - Personal responsibility → Superstitious behaviors	−0.05	−0.127	0.012
Illness coherence → Belief - Personal responsibility → RG-Honesty and control	0.00	−0.042	0.027
Illness coherence → Belief - Personal responsibility → RG-Precommitment	−0.01	−0.067	0.044
Illness coherence → Belief - Personal responsibility → Superstitious behaviors	0.00	−0.006	0.019
Emotional representations → Belief - Personal responsibility → RG-Honesty and control	−0.02	−0.056	0.011
Emotional representations → Belief - Personal responsibility → RG-Precommitment	−0.03	−0.086	0.019
Emotional representations → Belief - Personal responsibility → Superstitious behaviors	0.01	−0.002	0.028
*Indirect effects: Positive ambling belief - Gambling literacy as the mediator*			
Consequences → Belief - Gambling literacy → RG-Honesty and control	0.04^*^	0.009	0.075
Consequences → Belief - Gambling literacy → RG-Precommitment	0.02	−0.015	0.052
Consequences → Belief - Gambling literacy → Superstitious behaviors	−0.11^***^	−0.187	−0.058
Illness coherence → Belief - Gambling literacy → RG-Honesty and control	0.00	−0.010	0.022
Illness coherence → Belief - Gambling literacy → RG-Precommitment	0.00	−0.004	0.017
Illness coherence → Belief - Gambling literacy → Superstitious behaviors	−0.01	−0.052	0.035
Emotional representations → Belief - Gambling literacy → RG-Honesty and control	−0.03^*^	−0.055	−0.007
Emotional representations → Belief - Gambling literacy → RG-Precommitment	−0.01	−0.037	0.009
Emotional representations → Belief - Gambling literacy → Superstitious behaviors	0.08^**^	0.036	0.133

RG, responsible gambling. ^*^p < 0.05, ^**^p < 0.01, ^***^p < 0.001.

As displayed in Table 3 [(b) Mediation model], the indirect effects of perceived consequences on RG behaviors (both honesty-control and precommitment) were significant *via* beliefs about personal responsibility, with standardized indirect effect = 0.19 (*95% CI* [0.119, 0.267]), *p* < 0.001, and standardized indirect effect = 0.30 (*95% CI* [0.218, 0.400]), *p* < 0.001, respectively, supporting *H4*a. Its indirect effect on RG-honesty and control *via* gambling literacy belief was also significant, with standardized indirect effect = 0.04 (*95% CI* [0.009, 0.075]), *p* = 0.026, supporting *H4*b. However, only the indirect effect of perceived consequences on superstitious gambling behaviors *via* gambling literacy beliefs, but not personal responsibility, was significant, with standardized indirect effect = −0.11 (*95% CI* [−0.187, −0.058]), *p* = 0.001 and standardized indirect effect = −0.05 (*95% CI* [−0.127, 0.012]), *p* = 0.205, respectively, supporting *H4*d but not *H4*c. The indirect effect from emotional representations of GD to RG-honesty and control was significant (standardized indirect effect = −0.03, *95% CI* [−0.055, −0.007], *p* = 0.036), *via* gambling literacy as a full mediator, whereas the indirect effect of emotional representation on superstitious behaviors was also significant, with standardized indirect effect = 0.08 (*95% CI* [0.036, 0.133]), *p* = 0.002, *via* gambling literacy as a partial mediator. Therefore, *H6*b and *H6*d were supported. However, the *H6*a and *H6*c hypotheses regarding the indirect effect of emotional representations *via* personal responsibility belief on two types of RG behaviors and superstitious gambling behaviors (standardized indirect effect = −0.03 to 0.01, *p* = 0.200 to 0.387) were not supported.

## Discussion

Using a CSM framework to examine gambling-related beliefs and behaviors, our study was the first to explore the cognitive mechanisms underlying the potential influences of illness representations of GD on gambling behaviors. The findings of the current study not only demonstrated that illness representations of GD are associated with gamblers’ healthy and superstitious patterns of gambling but also revealed the mediating role of positive gambling beliefs in such associations.

As hypothesized (*H1*a and *H1*b), perceived consequences showed an overall positive effect on RG and a negative effect on superstitious gambling among Chinese adult gamblers. Although an early meta-analysis of 45 empirical studies utilizing CSM as a framework suggested that patients who perceived greater consequences of their illnesses were more likely to adopt avoidance coping strategies (Hagger and Orbell, 2003), more recent studies conducted among general patient populations and those with mental disorders have shown that perceived consequences of an illness were associated with higher levels of healthcare use, as well as help-seeking and active coping, respectively, with respect to physical diseases (Frostholm et al., 2005; Baines and Wittkowski, 2013; Richardson et al., 2017). The finding of this study added to the literature that this specific illness representation may be a salient factor for enhancing precautionary behaviors (e.g., controlled gambling in our case) and hindering vulnerable ones (e.g., superstitious gambling in our case) for behavioral addictions.

The results of mediation testing further revealed that perceived consequences most likely influenced those behaviors indirectly *via* promoting beliefs of positive gambling, in terms of taking self-responsibility to protect oneself from gambling-related harms and having a more accurate perception of the true nature of gambling, which is based on chance. Our corresponding hypotheses, *H4*a, *H4*b, *H4*c, and *H4*d, were all supported by SEM results, showing that the associations between perceived consequences of GD and gambling behaviors were fully mediated by positive gambling beliefs. These findings suggest that introduction or/and education about the severe consequences of GD may be considered in future programs that aim to promote RG in gamblers.

The findings of a previous systematic review supported a mild positive association of illness coherence with problem-focused coping (Richardson et al., 2017), and thus this illness representation may properly help individuals deal with the health threat. To our surprise, SEM results showed that illness coherence of GD was not significantly related to either positive gambling beliefs or RG behaviors. Furthermore, illness coherence was even found to be positively, instead of negatively, associated with superstitious gambling behaviors, although its indirect effects on responsible/superstitious gambling behaviors were found to be nonsignificant. Therefore, our testing failed to support all our hypotheses about illness coherence (i.e., *H2*a, *H2*b, *H5*a, *H5*b, *H5*c, and *H5*d). These findings are plausible because such perceptions of GD are the result of a subjective evaluation of self-knowledge about GD and thus may be susceptible to misinformation and self-serving biases. The potential discrepancy between the actual knowledge level of GD and the perceived level may explain the nonsignificant association between illness coherence of GD and positive gambling beliefs, including gambling literacy, in this study. Furthermore, given previous findings showing that the effects of illness coherence on outcomes (e.g., role functioning and disease state) are not mediated by problem-focused coping and that the association of illness coherence with mental wellbeing is stronger when compared to its association with problem-focused coping (Hagger et al., 2017; Richardson et al., 2017), we also speculate that one’s having a better and more coherent understanding of an illness may play a more prominent role in protecting one’s psychological wellbeing than it does in promoting preventive behaviors. Further research on the effects of perceived and actual illness understanding across multiple illnesses is warranted to test the aforementioned speculations. Moreover, qualitative studies may be called for to gain a clearer picture of gamblers’ understanding of GD.

The negative link between emotional representations of GD and RG behaviors (*H3*a) and the positive link between emotional representations of GD and superstitious gambling (*H3*b) were supported by our SEM results. These findings are congruent with the CSM framework, which proposes that strong emotional responses to a health threat will drive individuals to avoid dealing with the threat, which results in maladaptive coping behaviors and poor health outcomes (Leventhal et al., 1998, 2012). Indeed, the impeding effects of emotional representations of physical illnesses on various health behaviors (e.g., proactive coping like medication adherence) have been consistently reported in previous empirical studies (Kucukarslan, 2012; Hagger et al., 2017; Hagger and Orbell, 2021). Specifically, this study identified gambling literacy belief as a full and partial mediator of the effect of emotional representations of GD on responsible and superstitious gambling, supporting *H6*b and *H6*d, respectively. Our findings showed that strong negative emotional responses, such as fear and anxiety regarding GD, may hinder gamblers from developing gambling literacy, which leads to lower adherence to RG practices and more irrational (e.g., superstitious) behaviors during gambling.

In contrast, gamblers with greater levels of emotional representations of GD may directly utilize superstitious gambling to regulate negative emotions related to GD because individuals often cope with such negative emotions with emotion-focused strategies (Leventhal et al., 1998); moreover, superstition can, in fact, be an emotion-focused coping *per se* (García-Montes et al., 2008; Dömötör et al., 2016). However, emotional representations of GD were not significantly linked to beliefs in personal responsibility, at least among our Chinese adult gamblers, and *H6*a and *H6*c were not supported. Future research may further test the indirect effects of illness representations of GD on RG *via* other gambling beliefs (e.g., erroneous gambling beliefs) to explore the most useful type(s) of beliefs and relevant mechanisms that may inform more cost-effective RG interventions.

### Limitations

There are some limitations of this study that should be noted. First, causal relations among the psychological variables cannot be inferred based on our results, as a cross-sectional research design was adopted (Solem, 2015; Spector, 2019). We suggest that future researchers conduct a randomized control trial study to test whether enhancing gamblers’ perceived consequences would result in higher levels of positive gambling beliefs and RG adherence. We also recommend using longitudinal studies to test the potential reciprocal effects between illness representations of GD and gambling-specific beliefs. Second, the convenience sampling method may limit the generalizability of our current findings to all Chinese gamblers and gamblers of other ethnicities. To test the replicability of current findings, future researchers may want to involve other ethnic populations with a probability sampling method. Third, as a self-report survey, our results might be influenced by social desirability bias (Nederhof, 1985). We recommend that future research consider collecting data, particularly those related to gambling behaviors, from multiple sources (e.g., the information provided by participants’ family members or close friends) for cross-examination. Furthermore, CSM, the theoretical framework of this study, does not take individuals’ actual understanding of GD (e.g., the accuracy and extent of their knowledge of GD) into account. Future studies can compare some subjective perceptions of GD (i.e., illness representations defined in the current study) with its alternative objective form for further exploration.

### Implications and conclusion

Despite the aforementioned limitations, the current study has several notable theoretical and practical implications. First, we extended the application of CSM to controlled and at-risk gambling by empirically providing evidence that illness representations of GD, at least with respect to perceived consequences and emotional representations, are significantly associated with both responsible and superstitious gambling behaviors. Second, we provided some empirical support for the mediating role of gambling-specific cognitions (i.e., positive gambling beliefs in our case) on the relationship between GD representations and gambling behaviors. Such findings provide a theoretical framework for guiding future research, which may perhaps adopt a cognitive-behavioral perspective when evaluating the direct and indirect effects of different types of cognitions of GD, as well as gambling, on healthy and/or disordered gambling patterns. Third, the significant, direct effects of emotional representations of GD on superstitious behaviors suggest that superstitious gambling may serve as an emotional regulation strategy for gamblers, which provides new insights into gambling-related superstitions from an emotional perspective. Last but not least, the differential associations of personal responsibility and gambling literacy with both GD representations and gambling behaviors were revealed in our study for the first time, suggesting the necessity of further examining the potential diverse effects of individual gambling-specific beliefs across gambling behaviors in future studies.

Based on the current findings, we recommend that psychoeducation programs (e.g., in the form of educational videos; Hollingshead et al., 2019) be adopted in RG promotion campaigns in both the general public and gamblers to alter their illness representations (e.g., to heighten their awareness of the negative consequences of GD while lowering their negative responses to GD, which may be due to misunderstanding or stigma regarding people with GD) *via* providing better information about GD as a mental illness. Promoting more accurate knowledge about consequences of a disorder and weakening its negative emotion representation may also improve help-seeking (Hubbard et al., 2016; Yang et al., 2022). Moreover, future RG promotion campaigns may consider instilling positive gambling beliefs in gamblers because gambling beliefs (particularly gambling literacy) may promote gamblers’ RG while hindering their superstitious gambling, which may reduce their risk of developing GD, benefiting both the wellbeing of gamblers and the society in the long run.

## Data availability statement

The raw data supporting the conclusions of this article will be made available by the authors, without undue reservation.

## Ethics statement

The studies involving human participants were reviewed and approved by the research ethics committee of the Department of Psychology at the University of Macau (reference number: 2022-02). The patients/participants provided their written informed consent to participate in this study.

## Author contributions

AW: conceptualization, funding acquisition, methodology, supervision, coordination, and writing – reviewing and editing. HY: conceptualization, data collection, methodology, data analysis, finding interpretation, writing – original draft, and writing – reviewing and editing. HZ, LD, and JC: writing – reviewing and editing. All authors contributed to the article and approved the submitted version.

## Funding

This study received funding from [Education Fund of the Macao SAR Government] under grant agreement no. [HSS-UMAC-2021-09].

## Conflict of interest

The authors declare that the research was conducted in the absence of any commercial or financial relationships that could be construed as a potential conflict of interest.

## Publisher’s note

All claims expressed in this article are solely those of the authors and do not necessarily represent those of their affiliated organizations, or those of the publisher, the editors and the reviewers. Any product that may be evaluated in this article, or claim that may be made by its manufacturer, is not guaranteed or endorsed by the publisher.
